# Safe Sport in Mediterranean Contexts: Cultural Logics of Harm, Silence, and Authority

**DOI:** 10.1002/ejsc.70168

**Published:** 2026-04-15

**Authors:** Stiliani “Ani” Chroni, Eivind Å. Skille, Per Øystein Hansen, Antonis Alexopoulos, Juan de Dios Benítez‐Sillero, Ruth Brazier, Juan Calmaestra, Anna Kavoura, Renzo Kerr‐Cumbo, Sergio Lara‐Bercial, Alexander Navarro, Miguel Nery, Chiara Nicolini, Helena Verhelle, Sara Vivirito, Mary Hassandra

**Affiliations:** ^1^ Section of Sport and Physical Education University of Inland Norway Elverum Norway; ^2^ Department of PE & Sports Sciences University of Thessaly Trikala Greece; ^3^ European University Cyprus Engomi Cyprus; ^4^ Universidad de Córdoba Córdoba Spain; ^5^ Leeds Beckett University Leeds UK; ^6^ University of Ioannina Ioannina Greece; ^7^ MCAST Institute of Community Services Paola Malta; ^8^ Sport Coaching Europe Is‐Swieqi Malta; ^9^ International Council of Coaching Excellence Leeds UK; ^10^ Faculdade de Motricidade Humana Universidade de Lisboa Lisbon Portugal; ^11^ CEIPES ETS International Centre for the Promotion of Education and Development Palermo Italy; ^12^ Safeguarding Sport & Society Centre of Expertise Care and Well‐being Thomas More University of Applied Sciences Antwerp Belgium

**Keywords:** cultural logics, cultural praxis, interpersonal violence, safe sport, safeguarding

## Abstract

This study explores how cultural logics, as articulated by system‐level actors in the Northern Mediterranean region, may shape the acceptance, tolerance, and normalisation of interpersonal violence in sport, and constrain efforts to develop and implement safe sport initiatives. Using a cultural praxis heuristic, we conducted 60 semi‐structured interviews with individuals responsible for coach education and recruitment in Cyprus, Greece, Italy, Malta, Spain, and Portugal. Analysis identified three themes: patriarchal structures reinforcing hierarchical power dynamics and gender norms; a win‐focused culture prioritising competitive success over athlete well‐being; and cultures of blindness and silence through which harmful practices are overlooked, minimised, or contained. Across contexts, safeguarding was described as less salient than adjacent institutional priorities (e.g., doping and match‐fixing), and participants highlighted a tension between expectations for top‐down reform and mistrust toward institutional authorities, helping to explain why safeguarding remains weakly operationalised in everyday governance. Addressing IV therefore requires explicit attention to the cultural logics through which authority is exercised, success is evaluated, and concerns are handled within routine organisational practice. Implications include strengthening coach education and governance processes, supporting culture‐change efforts that rebalance performance and welfare priorities, and engaging local actors to increase accountability and voice.

## Highlights

- Cultural expectations around obedience, loyalty, and reputation shape how harm is recognised, minimised, or kept internal.
- Authority and performance priorities, institutionalised through recruitment, evaluation, and complaint practices, marginalise safeguarding and normalise containment of concerns.
- Advancing safe sport requires revisiting cultural norms, promoting athlete‐centred development‐focused sport experiences, and fostering community engagement.

## 1 Introduction

Interpersonal violence (IV) in sport is a serious and complex issue with profound consequences for athletes, families, coaches, and sport communities. The World Report on Violence and Health (WHO 2002), the new International Olympic Committee (IOC) Consensus Statement (Tuakli‐Wosornu et al. 2024) and the European Sport Psychology Federation Position Statement (Khomutova et al. 2025) describe IV as the intentional use of force or power against another individual, which can manifest in physical, sexual, psychological, or neglect‐based harm. These forms of violence can affect people across all ages, genders, ethnicities, and social backgrounds, leading to long‐term psychological distress, physical injuries, and socio‐economic challenges such as substance abuse, depression, suicidal ideation, and disrupted educational or professional opportunities (WHO 2014, 2020). Sport is an environment where IV can be overlooked, normalised, or inadequately addressed (Stirling and Kerr 2009), and where meaning‐making frameworks such as *Safe Sport* and *Safeguarding* emerged to mitigate IV and protect athletes from harm. Safe sport promotes physically and psychologically safe environments where participants can thrive, while safeguarding refers to proactive and holistic measures that prevent and respond to IV in sport (Tuakli‐Wosornu et al. 2024). The adoption and effectiveness of safe sport and safeguarding initiatives are shaped by cultural and societal factors, as harm and violence are understood, articulated, and acted on differently across contexts through culturally embedded discourses around authority, responsibility, and acceptable practice (Alexandre et al. 2022; Gurgis et al. 2023; Khomutova et al. 2025; Rhind and Owusu‐Sekyere 2020; Rulofs and Hartill 2025).

Here, we enquire into how cultural logics within Northern Mediterranean sport systems shape the understanding and enactment of safe sport, situating the region within a broader European landscape where research, policy development, and safeguarding measures addressing IV have evolved at different paces across contexts (Chroni and Kavoura 2022; Chroni and Papaefstathiou 2014; Hartill et al. 2024). To this day, psychological, physical, sexual violence, and neglect have been documented in sport (Mountjoy et al. 2016; Rulofs et al. 2019; Tuakli‐Wosornu et al. 2024), yet with varying prevalence as researchers use different conceptual and methodological approaches (e.g., Fasting et al. 2011; Parent and Vaillancourt‐Morel 2021; Vertommen et al. 2016). Definitions and manifestations of IV in sport are time‐ and place‐bound, impacting IV interpretations and the development of policies to address it (Chroni 2013; Lang and Hartill 2014).

Culture shapes shared values, influencing how people identify with each other and behave in specific contexts. It is transmitted formally and informally from generation to generation, forming the basis for daily behaviours and practices (Berger and Luckmann 1966). Ethical and social standards reflect the cultural context as understood by its members, an orientation aligned with principles of cultural relativism (Hahn 2023). Sports are characterised by unique cultures created through the blending of national, societal, sport‐specific, and local influences (E. Å. Skille and Chroni 2018). This blending of cultures has a role in how sport is conceptualised, approached, and performed from grassroots to the elite level and may help us understand how IV and initiatives against it are more or less accepted and managed (usually reactively) in different cultures. In this study, we attend to everyday practices and organisational routines that can normalise, obscure, or sustain IV within sport systems.

Culture has received some attention as an IV risk factor (Khomutova et al. 2025). Recent findings indicate that sport cultures in which winning at all costs is prioritised, outdated sport models that normalise abuse, and practices that neglect athletes' needs can prime sportspersons to accept and tolerate IV (e.g., Ohlert et al. 2021; Schmidt et al. 2022). For instance, the 2023 Women's Football World Cup medal ceremony incident involving the Spanish Football Federation president illustrated how culturally embedded interpretations of authority and intimacy can shape responses to alleged harm. Public debate revealed tensions between international norms and local framings of the president's conduct, which he defended as a ‘cultural misunderstanding’ and criticised subsequent reactions as ‘false feminism’ and ‘social assassination’ (Snape and Kassam 2023; Kassam 2023). This episode highlights how authority and culturally embedded meanings can render certain behaviours defensible or downplayed, even when they are publicly contested.

Research across sport sociology, sport psychology, and sport management demonstrates that shared cultural assumptions and institutional structures play a role in sustaining IV in sport. Drawing on Enfield's (2000) theory of cultural logic, we understand cultural logics as shared premises and taken‐for‐granted assumptions through which actors interpret actions, attribute intentions, and justify practices within their social worlds. In this sense, cultural logics do not refer to fixed cultural traits but to inferential frameworks reproduced through everyday interaction and institutional routines. Feminist sport scholarship has long situated organised sport within patriarchal power structures that normalise hierarchical authority and male‐dominated leadership (Vertinsky 2012). Within such contexts, coach‐athlete relationships are characterised by pronounced power asymmetries and expectations of obedience (Brackenridge 1997; Nite and Nauright 2020). Empirical studies further demonstrate that performance‐oriented climates and win‐at‐all‐costs logics intensify these hierarchies by aligning legitimacy with results and marginalising athlete voice (Ohlert et al. 2021; Greither and Ohlert 2024). Psychological maltreatment and other forms of harm are frequently normalised as necessary for success or commitment to the ‘sport ethic’ (McGee et al. 2024; Kerr et al. 2019). Recent Mediterranean region evidence indicates that psychological violence is perceived as less harmful than sexual or physical violence, though it is the most commonly observed form of IV in coaching contexts, further complicating recognition and response (Chroni et al. 2026). Such normalisation can be sustained through organisational practices and structures, including internal investigative processes, shielding of leadership, obscured reporting lines, valorisation of institutional actors, and the discouragement of formal documentation, that prioritise institutional reputation and internal cohesion over transparency and accountability (Nite and Nauright 2020).

A common practice among countries in addressing IV in sport has been the adoption of translated global safeguarding initiatives, like the *IOC Toolkit for IFs and NOCs* (Burrows 2017) or the European Union and Council of Europe's *Child Safeguarding in Sport* action guidelines (European Union and Council of Europe 2025). However, implementation appears to involve limited attention to cultural adaptations, raising concerns about the extent to which such frameworks adequately account for local norms around authority, reporting, and acceptable coaching behaviour. Recognising the importance of contextual sensitivity, the International Safeguarding Children in Sport Working Group (2016) emphasised the need for region‐specific research and tailored safeguarding strategies. Within the six countries studied here, research on IV remains limited and fragmented. Existing studies have primarily focused on athletes' experiences, particularly sexual violence and harassment experienced by women, documenting under‐reporting, normalisation of harmful practices, and constrained institutional responses (see Alexandre et al. 2022; Chroni and Fasting 2009; Chroni and Kavoura 2022; Hartill et al. 2024, 2023). There is limited in‐depth insight into how coach‐facing procedures (education, recruitment, supervision, complaint handling) translate contextual norms into everyday decisions about what counts as harm, associated practices, and whether concerns are pursued.

In the European Union, safe sport is a priority area (Council of the European Union 2024) and coaches are recognised as ‘first respondents’ in making environments safe and rendering positive sport experiences (Council of the and European Union 2017), with the recommendation to equip them with necessary knowledge, skills, and competencies (Council of the European Union 2020). However, coaches are themselves products of sport systems. Some have experienced IV during their athlete years and may reproduce or rework those experiences in their coaching practice. McMahon et al. (2020) report on a swimming coach who transferred into her coaching the physical and psychological abuse she had normalised as an athlete. Coaches' practices are closely linked to their histories and ‘the social interaction in which they are embedded’ (Jones and Corsby 2015, 442).

In contemporary safe sport discourse, coaches are often positioned primarily as potential risk factors or perpetrators of IV, with interventions focussing on changing individual coaches' knowledge, attitudes, and behaviours (e.g., Duda and Appleton 2016; Kerr et al. 2019; Tuakli‐Wosornu et al. 2024). However, coaches are educated, certified, hired, and supervised within sport systems that define what counts as ‘normal, effective, and/or good’ coaching, what is tolerated in the name of performance, and how (if at all) safeguarding responsibilities are enacted. These systems are themselves embedded in broader cultural logics and power relations (Ryba and Wright 2005). In this study, we therefore approached individuals who design and deliver coach education or are responsible for recruiting and employing coaches, recognising them as information‐rich sources of insight into the cultural assumptions, institutional priorities, and everyday practices that shape how safe sport is understood and enacted in their countries. By enquiring into the perspectives of such ‘gatekeepers’ across six Mediterranean countries, we examine cultural logics that may influence the acceptance, tolerance, and contestation of IV in sport, particularly within coach education, recruitment, and supervision systems. In this study, we examine how these frameworks become institutionalised in everyday practices of coach education, recruitment, supervision, and complaint handling.

Sociological and demographic scholarship has identified contrasts between Northern and Southern European societies in the organisation of family and social life, with southern contexts often characterised by relatively strong intergenerational ties and dense relational networks (Reher 1998). These characteristics provide contextual background for considering how authority, responsibility, and social cohesion may be organised within particular settings.

Recognising that coaches are educated, certified, hired, and supervised within culturally infused systems, we interviewed individuals responsible for coach education and recruitment to address the following research question: In what ways do cultural logics, as articulated by coaching gatekeepers across Cyprus, Greece, Italy, Malta, Spain, and Portugal, underpin the acceptance, tolerance, and normalisation of IV in sport, and shape efforts to advance safe sport? The grouping of Cyprus, Greece, Italy, Malta, Spain, and Portugal functioned as an analytic heuristic. Scholarship has identified recurring sociocultural patterns across Southern European contexts, including dense relational networks, intergenerational ties, and hierarchical authority structures (e.g., Chroni and Kavoura 2022; Muscat et al. 2020; Reher 1998), which provided contextual background for this study. However, we did not assume cultural homogeneity or fixed regional traits. Instead, we examined whether recurring patterns were reflected in how IV and safe sport are interpreted and enacted within coach education, recruitment, and supervision systems.

## 2 Methods

This study was guided by Ryba and Wright's (2005) cultural praxis heuristic and principles of cultural relativism (Berger and Luckmann 1966; Hahn 2023) to explore how cultural logics within six sport systems shape the understanding and enactment of IV and safe sport. In line with cultural praxis, we conceptualised cultural logics as patterned ways of organising meaning and authority that become visible through everyday practices and institutional routines (Enfield 2000). Cultural praxis critically challenges assumptions in sport, highlighting how cultural norms, values, power relations, and institutions shape lived experiences (Chroni and Kavoura 2020). From this perspective, sport cultures (and discourses around safe sport) are not static, but reproduced or resisted through daily practices and interactions. The cultural praxis heuristic enabled us to examine how local and global influences intersect in shaping safe, inclusive, and fair sport environments (Ryba 2017), while foregrounding participants' understandings of their contexts. This is also in line with the principles of cultural relativism (Hahn 2023), which deliberately avoids comparisons between countries. Cultural praxis and cultural relativism, including our conceptualisation of cultural logics, informed our conceptual framing, methodological decisions, and interpretation of the data.

### 2.1 Participants

A total of 60 semi‐structured interviews were conducted with individuals from (a) education settings that develop and deliver coaching curricula, for example, universities, federations, and (b) sport settings that hire coaches, for example, clubs, teams, federations. We deliberately targeted individuals responsible for coach education and for recruiting and/or supervising coaches as they occupy roles that shape how coaches are prepared, selected, supervised, and evaluated. They were therefore well positioned to reflect on institutional priorities, normative expectations, and everyday practices shaping coaches' responsibilities regarding safe sport. We aimed for representation of genders, acknowledging that women are not equally represented in sport leadership roles, and employed purposeful sampling to meet our criteria and conditions (Patton 2015). National researchers developed a pool of potential participants, using personal and professional networks, and invited them via phone calls and emails. Ten interviews were conducted per country. The equal distribution supported representation across contexts while allowing the identification of recurring patterns. Out of the 60 interviewees, 11 were from coach education settings and 49 from sport settings. Their roles covered grassroots and competitive sport environments. This facilitated a system‐level perspective on coach preparation and safe sport, aligned with our attention on system‐level cultural logics across performance levels. Gender‐wise, 13 identified as women and 47 as men. Table 1 presents additional participant information.

**TABLE 1:** Participant information for the 10 interviews conducted per country.

Country	*M*_*age*_	Men	Women	General education	Sport education	Roles of interviewees
Cyprus	48.0	9	1	9	8	National sport schemes officer, general manager of federation, officer of national stakeholder, federation president, sport club director/general manager, technical director, coaching course director, university educator
Greece	56.2	7	3	9	9	Coaches' association board member, head coach, university educator, referee educator, technical advisor, director of competitive sports, coach education and development, board member of federation
Italy	42.8	6	4	5	10	Team coordinator, coach, national coach trainer, coach educator, managing coaches, managing technical staff, team manager
Malta	44.7	10	0	8	5	Head of coach education, chief sport officer, university educator, CEO, directors of women's sport, club president, education officer, sports operations director
Spain	32.3	7	3	7	7	Federation strategic planning, coach, referee, trainer
Portugal	45.9	8	2	10	10	Sport manager, university educator, manager

### 2.2 Data Collection

Semi‐structured interviews were used to explore specific focal areas while allowing flexibility to pursue depth and contextual nuance in the conversations (Kvale and Brinkmann 2009). The interviews explored four focal areas:

- understanding of the term safe sport,
- coaches' credentials, screening, and education on safe sport,
- views of what is (and is not) harmful, values, beliefs, behaviours, customs, habits manifested in sport, and
- thoughts on how sport can become safer.

These areas were designed to elicit how safe sport is defined, enacted, and governed within participants' sport systems. Topic areas 1 and 2 explored how participants understand safe sport and IV and processes used to prevent harm within their sport systems. Topic areas 3 and 4 focused on beliefs, views and values related to harm, how safe sport responsibilities are enacted within organisations and what participants felt could be improved. These topics enabled participants to articulate system‐level expectations and the patterned norms and power relations shaping coaches' roles in relation to safe sport. Across these topics, interviewees were encouraged to reflect on how coaches are framed (formally and informally) within their organisations with respect to recognising harm, preventing IV, and enacting safe sport responsibilities. Though the interview guide did not include direct questions labelled as ‘culture’, participants frequently framed their responses in terms of broader societal norms, family practices, institutional traditions, and taken‐for‐granted ways of doing sport, which we treated, following a cultural praxis heuristic, as articulations of culturally embedded meaning systems rather than isolated individual opinions.

Because terms such as IV, abuse, harm, or harmful behaviour in sport can be understood differently across contexts, interviewers encouraged participants to describe the specific behaviours or situations they had in mind when speaking about harm or unacceptable practice. Participants typically referred to behaviours ranging from shouting, humiliation, and emotional neglect to inappropriate physical contact, excessive training loads, or disregard for athletes' well‐being. The interviews did not impose predefined boundaries between acceptable and unacceptable behaviour; instead, participants articulated these boundaries in ways that reflected the norms, practices, and expectations within their sport systems. Accordingly, in the findings presented below, we retain participants' own terminology, treating variation in language use as analytically meaningful rather than as conceptual inconsistency, consistent with our cultural praxis approach to meaning making.

The interview guide was developed by all authors in English (used in Malta) and translated into four languages (Greek for Cyprus and Greece, Italian, Spanish, and Portuguese). Upon piloting it in each country, small refinements were made to the probing questions. Prior to data collection, the research team met virtually to discuss the interview guide and protocol. One of the national researchers served as interviewer in their respective countries. Before an interview, participants answered a brief online questionnaire about themselves (i.e., gender, age, position, role, tasks) and their country's sport system (i.e., coach recruitment, screening for safe sport, hiring, safe sport education). Their answers helped interviewers prepare by providing contextual points to stimulate and enrich the conversation. The interviews lasted between 43.5 and 100 min and yielded 1032 single‐spaced pages of verbatim transcriptions.

### 2.3 Data Analysis

Guided by Ryba and Wright's (2005) cultural praxis heuristic, our analytic stance moved beyond describing explicit content to interpreting how participants' narratives revealed culturally embedded assumptions, social norms, and institutional logics shaping IV and safe sport, with attention to shared meanings and authority. Cultural praxis encouraged us to attend to meanings underlying participants' talk, the broader cultural discourses they drew on, and the ways power relations were produced or challenged through language (Chroni and Kavoura 2020). Accordingly, we analysed the data by interpreting patterns of meaning across interviews and situating them within the sociocultural contexts in which participants work. Cross‐country themes were developed only where patterns of meaning recurred across multiple national datasets. Country‐specific nuances were retained in the coding process and are reflected in illustrative quotations. This analytic approach ensured that themes captured shared cultural logics organising meaning and authority within sport systems, beyond individual opinions.

Data were analysed following Braun and Clarke (2019),  (2022) six phases of reflexive thematic analysis. The first two phases were conducted by national researchers in their native language. Upon familiarising with the data (Phase 1), the research team met virtually to discuss the standardisation of the analysis. Following this session, national researchers analysed their data at the semantic level and coded the explicit content (Phase 2). Each country's codes, accompanied by exemplary raw data quotations, were translated into English for the subsequent phases of the analysis. In phase three (generating initial themes), the first three authors (not involved with data collection) adopted a latent analytic approach, examining underlying assumptions, discourses, and authority relations across contexts (Braun and Clarke 2019; Silverman 2021) to appraise contextualised statements from different countries and consider how these related to cultural characteristics. Semantic coding ensured closeness to participants' language while latent analysis enabled cross‐contextual interpretation of cultural meaning systems. These researchers were experienced in the study of the intersection of national cultural features with sport cultures (Chroni and Kavoura 2022; Hansen et al. 2021; E. Å. Skille and Chroni 2018; E. Skille et al. 2020). They first worked independently and then met to discuss and refine interpretations of cultural patterns related to IV and safe sport. Subsequently, in phase four, the full research team held another virtual meeting to review and discuss the research themes where national researchers acted as critical friends. In phase five, the three first authors made refinements on the presentation of themes and sub‐themes, while in phase six the first two authors draughted the paper and all co‐authors acted as critical friends.

Consistent with cultural praxis, reflexivity was integrated throughout the analytic process. The authors' professional backgrounds in safe sport, coaching, sport pedagogy, sociology, and cultural sport psychology informed our interpretations, prompting continuous reflection on how our assumptions, experiences, and positionalities shaped theme development. Reflexive memos and team discussions were used to question early interpretations, challenge taken‐for‐granted assumptions, and ensure that cultural interpretations were grounded in the data.

### 2.4 Ethics and Rigour of Study

Given the sensitivity of the topic, the multinational character of the study, and the fact that some researchers knew participants, we adhered to both procedural ethics (i.e., institutional board approval) and relational ethics (Ellis 2007). The project's lead institution in Greece secured institutional board approval covering data collection across all six countries. In accordance with national regulations, Cyprus, Spain, and Portugal obtained additional ethical approvals. All interviewees signed informed consent prior to completing the pre‐interview questionnaire and were reminded before each interview of their rights (i.e., to decline questions, withdraw from the study, and were informed on data storage and usage). Quotations are identified using numeric codes from I#1 to I#60.

Co‐authors acted as ‘critical friends’ throughout the study to enhance rigour through reflexive dialogue and alternative interpretations (Smith and McGannon 2018). We offered to each other multicultural perspectives and engaged in reflective dialogues to ensure analytic decisions remained grounded in the research question and data.

## 3 Findings and Discussion

This section presents an integrated analysis of the empirical findings, situating them within and extending existing scholarship.

### 3.1 Framing Safe Sport in the Mediterranean Context

Across the six countries, interviewees described safe sport as unevenly understood and weakly embedded in everyday practice. It was commonly linked to adjacent priorities like injury prevention and integrity management. In five of the six countries it was described primarily in terms of physical protection rather than relational or psychological harm: ‘The first thing I think about is the prevention of injuries, to plan a training that protects athletes from getting injured’ (I#50). Others framed safe sport through integrity threats like doping and match‐fixing, emphasising formal policy and compliance mechanisms. As one interviewee said, ‘We call it child safeguarding. We have threats from betting and doping; for these, we organise seminars for coaches, footballers, and officials, and have specific policy’ (I#6). Only in one country did a majority describe safe sport as encompassing multiple forms of interpersonal violence, linking it to ‘everything related with the safety of everyone; sexual harassment, bullying, racial discrimination, sexism’ (I#4). The data reveal broader conceptualisations of safe sport that nonetheless continue to privilege bodily protection and integrity compliance as the primary institutionalised understandings of safety, while psychological, sexual, and neglect‐related harms hold more peripheral positions.

These understandings translate into organisational priorities centred primarily on physical risk management, institutional integrity, and coach regulation, such as the sport policies for betting and doping described by Interviewee 6. From a cultural praxis perspective, such framings are not merely administrative choices but reflect patterned ways of organising meaning within sport systems that privilege performance protection and compliance over relational accountability and athlete welfare. In line with previous research, they shape what counts as harm and what becomes routinised and normalised in everyday sport experiences. When safety is anchored primarily in bodily protection or integrity threats, psychological, sexual, and neglect‐related harms are rendered less visible and less urgent within institutionalised sport systems. This pattern suggests that safeguarding is more readily institutionalised through risk management and compliance‐oriented logics, while relational and psychological harms receive comparatively less structural attention within the sport systems examined (Brackenridge 1997; Stirling and Kerr 2009; Tuakli‐Wosornu et al. 2024).

Against this general landscape, three main themes were identified (see Table 2), demonstrating how patriarchal authority, performance priorities, and everyday practices of blindness and silence co‐produce the conditions under which IV and safe sport are interpreted and managed. As participants described overlapping patterns across countries in how authority, performance, and harm were understood and enacted, the themes represent patterned logics identified across interviews and institutional settings.

**TABLE 2:** Thematic map of study's analyses findings.

Themes	Sub‐themes
Patriarchal culture	Gendered hierarchies and coaching roles
	Normalisation of shouting and abusive authority
	Centralised power, dependence, and mistrust
Winning‐focused culture	Winning as an intense priority
	Cultivating athlete dependency
	Prioritising results in coach recruitment
Cultures of blindness and silence	Blindness and silence as mechanisms of loyalty and hierarchy
	‘Anything goes’ attitudes and the normalisation of harm
	Two‐sided concerns about reputation and retaliation

### 3.2 Patriarchal Culture

Interviewees described gendered and hierarchical expectations as structuring who is recognised as legitimate, whose authority is protected, and how concerns are handled within their sport systems. Leadership was routinely associated with masculine authority, seniority, and reputational status, while questioning established figures was portrayed as socially and professionally risky. These expectations were visible in what was shared about coach recruitment based on familiarity and standing, reluctance to formalise complaints against respected coaches, and the prioritisation of loyalty over procedural accountability. We interpret these as patterned expressions of patriarchal authority organising meaning and power within the sport systems studied. Three sub‐themes capture how patriarchal authority is organised and reproduced: gendered hierarchies in coaching roles, the normalisation of shouting and abusive authority, and centralised power combined with dependence and mistrust.

#### 3.2.1 Gendered Hierarchies and Coaching Roles

Participants described sport and coaching as male‐dominated spaces where authority and legitimacy are routinely attributed to male coaches, who are seen as the primary authority figures shaping athletes' development. Understandings of gender and sexuality were described as ‘conditioned by stereotypes, coming from the roots of beliefs that have become very old‐fashioned, but that people still have very deep inside’ (I#59). Within this framing, women were positioned as more appropriate for supportive roles, while men were more readily associated with leadership and expertise.

This pattern aligns with research showing that gendered organisational cultures in sport position men as natural leaders and gatekeepers of authority, while marginalising women and non‐normative men in coaching pathways (Knoppers et al. 2024; Norman 2010). From a cultural praxis perspective, such hierarchies reflect broader patterned expectations about who is considered legitimate, knowledgeable, and trustworthy within sport (Chroni and Kavoura 2020). Such expectations shape whose authority is protected, whose behaviour is scrutinised, and how safeguarding responsibilities are distributed, monitored, or deferred within organisational routines, shaping how IV concerns are handled in practice.

In the sport systems examined, authority was described as sustained through informal and relational practices that often outweighed formal procedures. Appointments relied on familiarity, reputational standing, and long‐standing community ties, with decisions shaped by who is “known” and trusted locally. Challenging senior male figures was described as carrying reputational and relational costs extending beyond the club, particularly within dense family and community networks. In such contexts, hierarchical structures narrowed the space for questioning authority, and concerns about psychological aggression, humiliation, or excessive control were less likely to be formalised as safeguarding issues when the coach was perceived as legitimate or successful. In this way, informal recognition practices intersected with gendered hierarchies to narrow the conditions under which authority could be questioned through formal safeguarding channels.

Participants further emphasised the central role of families and close‐knit communities in reproducing these norms. One interviewee noted, “parents being concerned to know everything … [like] if there was a gay coach in the club it would be a problem” (I#5). Such examples illustrate how heteronormativity and suspicion of difference are cultivated early and carried into sport. Gendered authority was therefore reinforced not only within sport organisations but also through broader community expectations that shape who is recognised as an appropriate coach and whose authority is publicly supported or quietly protected.

#### 3.2.2 Normalisation of Shouting and Abusive Authority

The second sub‐theme shows how families and sport environments socialised individuals to accept shouting and everyday uses of power as normal and legitimate expressions of authority. Participants consistently described shouting as a routine means of enforcing hierarchy. Even “highly educated people [are] screaming like hell at a referee and offending her” (I#38), contributing to “a culture where violence is very, very normalised” (I#59). In this context, shouting was framed as part of “normal” coaching and sport life, blurring the boundary between discipline and psychological violence. As one participant explained, “the abuse becomes more visual and auditory when they use loud language, side‐lining them [athletes] on purpose, [and] becoming overtly aggressive with them” (I#35).

Such practices were not described as random but as embedded in performance expectations, particularly when coaches “impose their expectations on the children they coach; indirectly saying that you have to win‐at‐all‐costs” (I#38). Interviewees linked these behaviours to patriarchal coach‐athlete dynamics, in which “male authority holds significant power and top‐down power structures reinforce the existing patriarchal norms” (I#21). Within this meaning system, harsh or controlling conduct was more readily interpreted as strong leadership than as psychological violence, reducing the likelihood that such behaviours would trigger safeguarding scrutiny.

Research on disempowering and authoritarian coaching climates similarly demonstrates associations with increased psychological harm and reduced athlete wellbeing (Greither and Ohlert 2024; Ohlert et al. 2022). Interviewee's #21 observation that these practices reflect “principles, values, leadership styles from which we do not want to deviate” illustrates how such authority norms are enacted and defended. When shouting and control are normalised in this way, safeguarding initiatives can be perceived as unnecessary, intrusive, or misaligned with established coaching traditions. From a cultural praxis perspective, the normalisation of shouting operates as a patterned way of organising authority in everyday coaching practice, through which emotional pressure and control are rendered meaningful, legitimate, and resistant to external regulation.

#### 3.2.3 Centralised Power, Dependence, and Mistrust

The third sub‐theme shows how hierarchical structures are reinforced through centralised governance structures that concentrate authority and shape expectations about where change should originate. Participants described national federations and competitive structures as “characterised by both centralisation and top‐down structure, and an increased degree of intervening by the State and the Federations” (I#1). In this system, decision‐making authority was perceived as residing primarily with senior bodies, creating financial and organisational dependencies throughout the system. As a result, reform, including safe sport initiatives, was expected to come “from above.” One interviewee explained that change should “work from top to bottom, from the ministry to the coaches … I don't think the coaches should go out and rally for something they don't even know it exists” (I#11). This framing positioned coaches, athletes, and local administrators as recipients (not initiators) of safeguarding efforts, limiting bottom‐up challenge and reducing the likelihood that concerns would be raised independently within everyday coaching environments.

At the same time, participants described pervasive mistrust in these institutions. As Interviewee #3 noted, “it's not only in sports; there's lack of trust toward institutions in all areas of society. Because of this lack of trust, the ratification [of safe sport policy] will not be successful.” This combination of reliance on senior authorities for reform alongside scepticism about their integrity or effectiveness creates a paradoxical dynamic in which safeguarding is symbolically endorsed. Safeguarding is symbolically endorsed at the policy level yet weakly activated in practice, with reporting mechanisms underused and harmful behaviours left unchallenged (Lang and Hartill 2014; Wilson et al. 2022). From a cultural praxis lens, centralisation operates as a patterned way of organising authority in which responsibility is vertically allocated and accountability diffused. By concentrating decision‐making power while discouraging lateral or bottom‐up intervention, this governance logic contributes to the reproduction of existing hierarchies and constrains the everyday enactment of safeguarding within coach education, recruitment, and supervision systems.

#### 3.2.4 Cultural Meaning of Patriarchal Logics

Across the three sub‐themes, patriarchal authority emerges as a cultural logic organising meaning and power within the sport systems examined, particularly in contexts where prior scholarship has identified dense relational networks, strong intergenerational ties, and culturally embedded expectations of loyalty and authority in Southern European settings (Reher 1998; Chroni and Kavoura 2022; Muscat et al. 2020). Gendered hierarchies shape who is recognised as legitimate and whose authority is shielded from scrutiny; shouting and emotional control are normalised as credible expressions of leadership; and centralised governance structures position reform as hierarchically controlled rather than collectively enacted.

These dynamics structure how IV is interpreted, tolerated, and addressed within sport environments. They function as patterned structures reproduced through everyday practices in coach recruitment, status attribution, performance evaluation, complaint handling, and the routine enactment of coaching in training and competition settings. From a cultural praxis perspective, patriarchal authority therefore operates as a structuring logic that stabilises hierarchies, diffuses accountability, and narrows the pathways through which safeguarding concerns can be formally activated. Under such conditions, safeguarding initiatives risk remaining symbolic, as authority relations embedded in daily sport practice remain largely insulated from challenge.

### 3.3 Winning‐Focused Culture

The second theme, a strong emphasis on winning, was evident across the six countries. Although centred on performance, this emphasis was closely intertwined with hierarchical authority structures, centralised decision‐making, and broader expectations regarding obedience and respect. Interviewees framed winning as a societal expectation that legitimises particular coaching approaches and shapes how safeguarding is prioritised. Athlete welfare was described as negotiable when it was perceived to conflict with competitive success. This pattern aligns with research showing that performance‐oriented environments can normalise harmful coaching practices and reduce attention to athlete welfare (Kerr et al. 2019). The present findings show how this emphasis shapes everyday decisions about coach selection, authority, and safeguarding priorities within the sport systems examined. Three interrelated dynamics illustrate how winning operates as a cultural logic within these systems: winning as an intense priority, the cultivation of athlete dependency, and the prioritisation of results in coach recruitment. While performance‐oriented cultures are not unique to Southern Europe, the analysis of these dynamics shows how, in the sport systems studied, performance priorities intersect with centralised governance and loyalty‐based relational norms in ways that constrain safeguarding.

#### 3.3.1 Winning as an Intense Priority

Interviewees described winning as a deeply rooted societal priority that influences how safe sport is perceived and discussed. As I#27 noted, “people only think about winning … they care only about results and that affects how they see safe sport, as something less important.” This framing was portrayed as a shared expectation embedded in everyday sport conversations and decision‐making, where safeguarding was considered relevant only after performance concerns had been addressed or when a crisis emerged.

Participants linked this prioritisation of results to concrete decisions. Coaches were evaluated primarily on competitive outcomes, recruitment favoured those with demonstrable success records, and safeguarding concerns were described as secondary unless they threatened public reputation or performance stability. This marginalisation was described explicitly in some contexts, where safeguarding regulations were characterised as existing ‘on paper only’. “Most stakeholders consider other issues to be much more important than safe sport, although some sports clubs have regulations, these are “on paper only”, due to most stakeholders having a winning at all cost mentality, even in youth sports” (I#44). In such contexts, questioning intensive training regimes, emotional pressure, or dependency dynamics was framed as undermining competitive ambition. Research has shown that excellence‐oriented cultures tend to marginalise concerns about psychological safety and athlete well‐being, particularly when success is framed as the primary measure of values (Ohlert et al. 2022; Wilson et al. 2022). In the contexts studied, winning functioned as an organising criterion in recruitment, evaluation, and resource allocation, narrowing the institutional space in which athlete welfare concerns could be raised or pursued.

#### 3.3.2 Cultivating Athlete Dependency

Interviewees described the cultivation of athlete dependency on the coach, particularly within competitive and high‐performance pathways, as shaped by system‐wide norms. Athletes were portrayed as expected to trust the coach's judgement above all else and to accept behaviours that might cause harm. As I#17 shared, “many examples of coaches who adopted the authoritarian and violent style of leadership … I've seen slaps and I've been slapped … in front of the federation president … and they didn't do anything to the coach.” The absence of intervention, even in the presence of senior officials, shows how authority linked to performance could become insulated from scrutiny.

In systems characterised by respect for authority and loyalty to the “in‐group” (Chroni and Kavoura 2022; Muscat et al. 2020), dependency appeared intensified. Expectations of compliance and loyalty limited opportunities to question harmful practices. Even high‐performing athletes were described as needing protection, “these girls… have received international medals… [and] need to be taken care of” (I#22), highlighting that success did not shield athletes from unsafe environments.

Dependency also enabled harmful practices like “slaps” to be reframed as “tough coaching/love” when linked to competitive results. As one interviewee explained, “it is understandable that the coach can do whatever he wants: when you need the knowledge of the coach to win, you accept the power of the coach” (I#8). This framing illustrates how performance dependency legitimises authority and narrows the conditions under which coaching behaviour is questioned, resonating with research showing that athletes may normalise harmful treatment as necessary for achievement when embedded within win‐oriented cultures (Chroni 2015; Coker‐Cranney et al. 2018; Roberts et al. 2020). Across the sport systems studied, this normalisation was reinforced through organisational routines and relational hierarchies that protected authority and limited scrutiny.

#### 3.3.3 Prioritising Results in Coach Recruitment

Winning‐focused values were institutionalised through recruitment practices shaping how coaches were selected, evaluated, and retained. Interviewees explained that organisations often prioritised coaches with proven win‐records, regardless of interpersonal conduct or safeguarding competence. As I#3 said, “the boards prefer coaches that put winning above all, rather than coaches who try to give a chance to all kids to play.” Competitive results were positioned as the primary indicator of legitimacy, implicitly downgrading relational and safeguarding expertise.

Recruitment was frequently described as informal and network‐based. Even where formal procedures existed, “nobody really checks” (I#2) credentials in practice; familiarity and perceived ability to deliver competitive results carried greater weight. Such practices reflected relational norms of loyalty and seniority alongside hierarchical decision‐making structures, limiting scrutiny of coaching behaviour. In this context, safeguarding competence became peripheral to established reputations and existing power relations.

Research on organisational cultures in sport shows that performance metrics often overshadow ethical considerations (McGee et al. 2024). From a cultural praxis perspective, these recruitment practices reproduce meanings of success that privilege performance over relational care and accountability.

#### 3.3.4 Winning as Cultural Justification for Harm

Across the three sub‐themes, winning functioned as an organising principle shaping authority, dependency, and institutional decision‐making. Safeguarding concerns were repeatedly positioned as secondary when competitive outcomes were at stake. The absence of intervention in the face of aggressive behaviours, and recruitment practices privileging win records over safeguarding competence, illustrate how performance criteria were institutionalised and reproduced existing hierarchies. Within this context, winning operated as a cultural justification for harm. Authoritarian coaching was reframed as commitment, dependency interpreted as trust, and exclusionary recruitment rationalised as necessary for maintaining competitive standards. Challenging high‐status coaches risked being interpreted as disloyal or anti‐performance, increasing the social cost of disclosure.

International research similarly documents how performance‐driven environments blur the boundary between demanding coaching and IV (Greither and Ohlert 2024; Kerr et al. 2019). The present findings show how this blurring was embedded in everyday organisational practices across the six sport systems examined, where power dynamics and relational loyalty intensified the alignment between performance incentives and authority. In this sense, winning‐focused culture operated as a system‐level cultural logic aligning performance incentives with existing power hierarchies, thereby constraining safe sport implementation.

### 3.4 Cultures of Blindness and Silence

Interviewees described a recurring tendency to handle concerns internally, informally, and discreetly rather than through formal safeguarding channels. Harmful behaviours were often reframed as misunderstandings, performance tensions, or private matters to be resolved within the club or federation. Reporting was described as rare unless incidents became publicly visible or reputationally damaging. These practices were justified through references to loyalty, protection of the organisation, and avoidance of scandal. Silence therefore operated not as passive ignorance but as an active organisational routine that filtered which concerns were escalated, which were contained, and which were normalised. Three interrelated dynamics illustrate how this cultural logic operates: loyalty to hierarchy and in‐group protection, permissive minimisation, and concerns about reputational damage and retaliation.

#### 3.4.1 Blindness and Silence as Mechanisms of Loyalty and Hierarchy

Interviewees described learning not to recognise certain behaviours as problematic, adopting everyday practices of looking away, minimising, or reframing incidents as “normal” aspects of sport. Harm was at times acknowledged abstractly yet denied in concrete situations: “I didn't know, I didn't see, I didn't hear anything” (I#1). As another participant noted, “We just look at the case that happens on the news, we say ‘Oh poor guy’ and then everyone forgets about it and we continue the same” (I#3). These conversations illustrated how blindness operates as selective recognition, where awareness does not translate into action.

Silence was described as a complementary mechanism. Speaking up was portrayed as risky, shameful, or socially inappropriate: “Everyone knows these things happen, but no one talks about it. There's a fear that speaking up will bring shame to the club or the team” (I#29). Silence functioned as a relational strategy for preserving cohesion and demonstrating loyalty. The same interviewee insisted about how “people hesitate to speak out … contributing to a culture of silence on issues of violence or abuse” (I#29). In this sense, silence operates as a protective mechanism, sustained through fears of exclusion, reputational damage, and violating community norms; elements reported also by Johansson (2022) and Roberts et al. (2020).

Together, blindness and silence reinforced hierarchical authority structures. When incidents were minimised or contained internally, “what happens at home [team, club] does not go public”, authority figures were shielded from scrutiny and concerns were less likely to reach formal safeguarding channels. In socially dense organisational settings where loyalty, familiarity, and reputation management were described as highly valued (Muscat et al. 2020), challenging senior figures risked social and relational consequences. As a result, incidents were often managed informally, reframed as misunderstandings, or left unaddressed to preserve cohesion, reputation, and existing status hierarchies. Such patterns have been identified in research showing that IV persists where cultural norms discourage questioning authority or disrupting group cohesion (Nite and Nauright 2020; Seanor et al. 2023).

#### 3.4.2 ‘Anything Goes’ Attitudes and the Normalisation of Harm

The second sub‐theme captured what participants described as a relaxed or permissive attitude, “anything goes,” “no problem,” or “let it be, have [a drink]”, used to downplay or dismiss harmful behaviours. As I#40 put it,

I think [there’s] another type of mentality, possibly Mediterranean, not just [country], given that we’re so relaxed on things that *anything goes*. ‘No problem, no problem, we’ll solve it!’ Whereas if you go to Central Europe, Northern Europe, no way you’ll get away with some stuff that happens here.

This comparison reflects the participants' own framing of contextual differences. Blindness was described as operating through minimisation and contextual reframing, whereby harmful behaviours were rendered unremarkable, humorous, or culturally expected, allowing violence to persist without being recognised as such. As a result, behaviours that might otherwise trigger formal investigation were treated as minor, solvable, or culturally typical, reducing the likelihood that safeguarding structures would be mobilised.

Participants further described this minimisation as normalised through humour, casual tolerance, and the assumption that issues will resolve on their own. Research suggests that such leniency can enable coercive practices, discrimination, and abuse to persist unchallenged (Greither and Ohlert 2024; McGee et al. 2024). These attitudes reflect subordination as a learnt practice, where confronting authority or calling out harm is perceived as more socially risky than enduring it, aligning with what Coker‐Cranney et al. (2018) describe as cultures of “endurance”.

#### 3.4.3 Two‐Sided Concerns About Reputation and Retaliation

This sub‐theme focuses on situations in which harm is recognised but not disclosed, driven by fears about reputational harm for both the accused and the accuser. Interviewees explained that people hesitated to report because they feared falsely accusing a coach, “a good judgement that does not destroy someone's life instantly, because someone made a false testimony to attack a coach” (I#47), and because they worried about personal consequences, such as losing playing opportunities, “a player is afraid to lose their place in the team … they know that if they speak probably no other team will want them” (I#33). These conversations suggest that silence was operated through a weighing of relational and career risks, where the perceived costs of speaking up exceed the anticipated benefits, reinforcing reliance on informal resolution and discouraging engagement with formal safeguarding pathways. Similar concerns have been documented in research on bystander inaction and fear of retaliation or career damage (Chroni 2015; Wilson et al. 2022). In the sport contexts examined, where personal reputation and relational ties were described as carrying significant cultural weight (Chroni and Kavoura 2022; Muscat et al. 2020), these fears appeared intensified.

Interviewees also highlighted mistrust in institutional mechanisms: “people are afraid to open‐up and report, and there is a mistrust toward authorities” (I#4). When reporting lines were unclear, safeguarding roles were poorly defined, or complaints were managed by individuals closely connected to those accused, reporting was perceived as dangerous, futile, or both. Previous research similarly shows that weak or inconsistently enforced safeguarding structures contribute to systemic silence, enabling harmful practices to continue unchecked (Hartill et al. 2023; Parent and Vaillancourt‐Morel 2021). Emerging research on preformative compliance in sport governance further suggests that safeguarding policies may be formally adopted yet weakly integrated into everyday organisational practices (Gomez and Schoch 2026).

#### 3.4.4 Cultural Logics of Blindness and Silence and Their Implications

Across these sub‐themes, blindness shaped what is recognised as harmful, and silence shaped whether recognised harm was escalated or contained. Together, they operated as patterned ways of organising meaning and authority within sport systems. Loyalty, hierarchy, reputation management, and relational harmony structured interpretations of harm and regulated disclosure practices, intersecting with patriarchal authority and performance priorities identified earlier. International research similarly shows how cultural norms, power imbalances, institutional mistrust, and cohesion norms sustain harmful sport environments (Greither and Ohlert 2024; Stirling and Kerr 2009).

Through a cultural praxis lens, these dynamics function as institutionalised routines rather than isolated behaviours. By privileging informal, relational management over formal escalation, cultures of blindness and silence stabilise existing authority patterns and diffuse accountability. In this way, silence operates as a systemic condition through which IV may persist without formal acknowledgement or institutional disruption.

### 3.5 Integrative Cultural Interpretation Across Themes

Patriarchal authority, winning priorities, and everyday practices of blindness and silence operate together in shaping how IV is interpreted, justified, and managed within the sport systems examined. These logics are visible in routine processes of coach education, recruitment, performance evaluation, and complaint handling. Across the analysis, recurring expectations emerged: senior coaches should not be openly challenged; protecting the club's reputation takes precedence over public disclosure; and competitive success legitimises demanding or controlling coaching practices. Authority is treated as natural and difficult to question. Harsh treatment is reframed as commitment to performance. Loyalty is prioritised over accountability. Behaviours that may constitute psychological harm are reinterpreted as discipline, leadership, or necessary toughness in pursuit of results. Harm is rarely sustained through explicit endorsement; rather, consistent with research on abuse embedded in institutional power relations (Brackenridge 1997; Nite and Nauright 2020), it is reproduced through everyday organisational routines that stabilise shared premises about authority, performance, and loyalty (Enfield 2000).

The intersection of patriarchal hierarchies and performance imperatives is central to this dynamic. The elevation of male coaches as legitimate authority figures intersects with win‐oriented evaluation systems that position results as the primary marker of competence. When authority and results align, scrutiny diminishes. Blindness and silence reinforce this protection by shaping what is recognised as harmful and whether recognised harm is escalated. Disclosure is filtered through loyalty to the in‐group, concern for reputational damage, and mistrust toward institutions. As a result, incidents are minimised, resolved informally, or left unreported, and silence operates as an organisational practice embedded in everyday decision‐making.

From a governance perspective, recognising the impact of how authority and responsibility are distributed in daily practice is imperative. Centralised expectations of reform, informal recruitment networks, result‐driven evaluations, and internal handling of complaints collectively protect established hierarchies and render safeguarding secondary. Effective safeguarding requires attention to how legitimacy is granted, how authority is exercised, and how accountability is enacted within coach education and oversight systems. New policies or training materials alone are not a solution.

This interpretation extends prior knowledge in two ways. While earlier scholarship has shown that harm in sport is linked to power asymmetries and performance climates (Brackenridge 1997; Kerr et al. 2019; Ohlert et al. 2022), the present findings illustrate how these dynamics are sustained through everyday governance practices in coach recruitment, evaluation, supervision, and complaint handling. In addition, by focussing on system‐level gatekeepers, the study shows how institutional routines normalise and protect harmful practices. In doing so, it complements research identifying gaps between safeguarding policy and lived practice (Lang and Hartill 2014; Hartill et al. 2023) by explaining why such gaps persist.

These findings respond directly to our research question by identifying the cultural logics that underpin the acceptance, tolerance, and normalisation of IV in sport. The three logics operate through shared expectations and routine organisational practices. While these logics have been identified in other sport contexts, the present analysis shows how they intersect and are enacted within the organisational and relational dynamics described by participants across the six countries studied. Intervening on individual awareness alone is therefore insufficient. Safeguarding challenges in the contexts examined are systemic rather than episodic: they are embedded in how sport organisations define authority, success, and responsibility, and in how these definitions are enacted in everyday practice. As these logics are reproduced through daily decisions in education, recruitment, and governance, they function as shared premises that guide interpretation and action within sport systems.

### 3.6 Conclusion, Limitations, and Implications

This study suggests that efforts to advance safe sport may remain limited if the cultural conditions that shape how harm is recognised, tolerated, or silenced are not addressed. Across interviews conducted in six countries, we identified three interrelated cultural logics, patriarchal authority, winning priorities, and cultures of blindness and silence, that structure everyday sport practices and constrain safeguarding efforts. These logics reinforce one another, creating environments in which IV may be normalised, minimised, or rendered invisible.

This study contributes to safe sport scholarship by providing a culturally situated explanation of how safeguarding may remain structurally marginal in sport systems. By offering empirical insight from Northern Mediterranean contexts, where system‐level qualitative research remains comparatively limited, the findings broaden the geographical scope of safe sport scholarship and provide context‐sensitive evidence of how cultural expectations shape safeguarding in practice. In doing so, it builds on prior work documenting the role of power asymmetries and performance cultures in sustaining harm (Kerr et al. 2019; McGee et al. 2024; Tuakli‐Wosornu et al. 2024), while demonstrating how these dynamics are embedded and reproduced within institutional routines.

Several limitations should be acknowledged. The regional focus limits transferability to other geopolitical contexts. Semi‐structured interviews may have been influenced by social desirability, hesitancy to discuss sensitive issues, or institutional loyalty. The underrepresentation of women limited the breadth of gendered perspectives, and translation processes may have reduced nuance in some participants' expressions. Furthermore, while participants' roles spanned grassroots and competitive environments, the analysis focused on system‐level cultural logics rather than differentiating performance levels. Future research should examine how these logics may manifest differently across performance levels and organisational layers.

The findings suggest that addressing IV in sport requires more than formal policy adoption or individual behaviour change. Without attending to the cultural logics that regulate authority, legitimise performance‐driven pressure, and shape disclosure practices, safeguarding risks remaining symbolic. Cultural logics operate through everyday organisational decisions concerning who is selected, rewarded, protected, or challenged, and need to be addressed within governance and coach development structures. Within these systems, coaches occupy a structurally central position as actors working with expectations that reward results, normalise hierarchy, and regulate disclosure. Coach education, recruitment, and supervision represent key aspects, where existing logics could be disrupted.

Implications emerging from this analysis include:

- Education initiatives that explicitly address cultural norms sustaining silence and normalisation of harm, enabling athletes, coaches, parents, and administrators to recognise and challenge harmful practices.
- Organisational strategies that rebalance performance metrics with athlete welfare indicators, strengthen gender equity in leadership, and increase transparency in complaint procedures.
- Community‐level engagement that involves families and local sport actors in reshaping norms around authority, voice, and accountability.

If cultural dynamics remain unexamined, efforts to address harm risk remaining superficial. Culturally informed approaches attending to how authority, legitimacy, and success are defined and enacted in sport are more likely to support meaningful and sustained change.

## Conflicts of Interest

The authors declare no conflicts of interest.

## Data Availability

Research data are not shared.

## References

[ejsc70168-bib-0002] Alexandre, J. , C. Castro , M. Gama , and P. Antunes . 2022. “Perceptions of Sexual Abuse in Sport: A Qualitative Study in the Portuguese Sports Community.” Frontiers in Sports and Active Living 4: 838480. 10.3389/fspor.2022.838480. 35813054 PMC9260509

[ejsc70168-bib-0003] Berger, P. , and T. Luckmann . 1966. The Social Construction of Reality. Penguin Book.

[ejsc70168-bib-0004] Brackenridge, C. 1997. “‘He Owned me Basically…’. Women’s Experience of Sexual Abuse in Sport.” International Review for the Sociology of Sport 32, no. 2: 115–130. 10.1177/101269097032002001.

[ejsc70168-bib-0005] Braun, V. , and V. Clarke . 2019. “Reflecting on Reflexive Thematic Analysis.” Qualitative Research in Sport, Exercise and Health 11, no. 4: 589–597. 10.1080/2159676X.2019.1628806.

[ejsc70168-bib-0006] Braun, V. , and V. Clarke . 2022. “Conceptual and Design Thinking for Thematic Analysis.” Qualitative Psychology 9, no. 1: 3–26. 10.1037/qup0000196.

[ejsc70168-bib-0008] Burrows, K. 2017. “Safeguarding Athletes From Harassment and Abuse in Sport: IOC Toolkit for Ifs and Nocs.” https://olympics.com/ioc/safe‐sport/assistance‐for‐olympic‐movement‐stakeholders.

[ejsc70168-bib-0009] Chroni, S. 2013. “Sexual Harassment of Female Athletes: One More Call for Safety.” In Gender and Sport: Challenges and Changes, edited by G. Pfister and M. K. Sisjord , 174–188. Waxman Publishing Co.

[ejsc70168-bib-0010] Chroni, S. 2015. “Sexual Exploitation in Women's Sport: Can Female Athletes Respond to It?” In Elite Sport and sport‐for‐all: Bridging the Two Cultures?, edited by R. Bailey and M. Talbot , 119–133. Routledge.

[ejsc70168-bib-0011] Chroni, S. , and K. Fasting . 2009. “Βιώματα σεξουαλικής παρενόχλησης από άνδρες σε αθλήτριες στην Ελλάδα [Prevalence of Male Sexual Harassment Among Female Sports Participants in Greece].” Inquiries in Sport and Physical Education 7, no. 3: 288–296. 10.26253/heal.uth.ojs.ispe.2009.1333.

[ejsc70168-bib-0012] Chroni, S. , M. Hassandra , H. Verhelle , et al. 2026. “Coach‐Perpetrated Violence: Witnessing, Perceived Harmfulness and the Role of Coaching Motivational Climate.” European Journal of Sport Science 26, no. 1: e70113. 10.1002/ejsc.70113. 41478840 PMC12757192

[ejsc70168-bib-0013] Chroni, S. , and A. Kavoura . 2020. “Cultural Praxis.” In The Routledge International Encyclopedia of Sport and Exercise Psychology, edited by D. Hackfort and R. J. Schinke , 227–238. Routledge.

[ejsc70168-bib-0014] Chroni, S. , and A. Kavoura . 2022. “From Silence to Speaking up About Sexual Violence in Greece: Olympic Journeys in a Culture That Neglects Safety.” Frontiers in Psychology 13: 862450. 10.3389/fpsyg.2022.862450. 35465498 PMC9020591

[ejsc70168-bib-0015] Chroni, S. , and M. Papaefstathiou . 2014. “Safeguarding and Child Protection in Sport in Two Southern European Countries: Greece and the Republic of Cyprus.” In Safeguarding, Child Protection and Abuse in Sport, edited by M. Lang and M. Hartill , 58–67. Routledge.

[ejsc70168-bib-0016] Coker‐Cranney, A. , J. C. Watson , M. Bernstein , D. K. Voelker , and J. Coakley . 2018. “How far is Too Far? Understanding Identity and Overconformity in Collegiate Wrestlers.” Qualitative Research in Sport, Exercise and Health 10, no. 1: 92–116. 10.1080/2159676X.2017.1372798. 30197830 PMC6124497

[ejsc70168-bib-0017] Council of the European Union 2017. Conclusions of the Council and of the Representatives of the Governments of the Member States, Meeting Within the Council, on the Role of Coaches in Society. C423/04. Official Journal of the European Union. https://eur‐lex.europa.eu/legal‐content/EN/TXT/PDF/?uri=OJ:C:2017:423:FULL&from=BG.

[ejsc70168-bib-0018] Council of the European Union . 2020. “Conclusions of the Council and of the Representatives of the Governments of the Member States Meeting Within the Council on Empowering Coaches by Enhancing Opportunities to Acquire Skills and Competences.” Official Journal of the European Union C196/01. https://eur‐lex.europa.eu/legal‐content/EN/TXT/PDF/?uri=CELEX:52020XG0611(01.

[ejsc70168-bib-0019] Council of the European Union . 2024. Resolution of the Council and of the Representatives of the Governments of the Member States Meeting Within the Council on the European Union Work Plan for Sport (2024‐2027). Official Journal of the European Union: C/2024/3527. https://eur‐lex.europa.eu/eli/C/2024/3527/oj/eng.

[ejsc70168-bib-0020] Duda, J. L. , and P. R. Appleton . 2016. “Empowering and Disempowering Coaching Climates: Conceptualization, Measurement Considerations, and Intervention Implications.” In Sport and Exercise Psychology Research, edited by M. Raab , P. Wylleman , R. Seiler , A.‐M. Elbe , and A. Hatzigeorgiadis , 373–388. Academic Press.

[ejsc70168-bib-0021] Ellis, C. 2007. “Telling Secrets, Revealing Lives: Relational Ethics in Research With Intimate Others.” Qualitative Inquiry 13, no. 1: 3–29. 10.1177/1077800406294947.

[ejsc70168-bib-0022] Enfield, N. J. 2000. “The Theory of Cultural Logic: How Individuals Combine Social Intelligence With Semiotics to Create and Maintain Cultural Meaning.” Cultural Dynamics 12, no. 1: 35–64. 10.1177/092137400001200102.

[ejsc70168-bib-0023] European Union & Council of Europe . 2025. Child Safeguarding in Sport, 2020‐2022. https://pjp‐eu.coe.int/en/web/pss.

[ejsc70168-bib-0024] Fasting, K. , S. Chroni , S. E. Hervik , and N. Knorre . 2011. “Sexual Harassment in Sport Toward Females in Three European Countries.” International Review for the Sociology of Sport 46, no. 1: 76–89. 10.1177/1012690210376295.

[ejsc70168-bib-0027] Gomez, C. , and L. Schoch . 2026. “Safeguarding in Sport: Toward a Performative Compliance of International Sports Federations?” International Review for the Sociology of Sport: 1–22. 10.1177/10126902251404389.

[ejsc70168-bib-0028] Greither, T. , and J. Ohlert . 2024. “Empowering and Disempowering Climate and Experiences of Psychological Violence in Artistic Gymnastics.” German Journal of Exercise and Sport Research 54, no. 4: 576–586. 10.1007/s12662-023-00886-7. PMC10266307 40479099

[ejsc70168-bib-0029] Gurgis, J. J. , G. Kerr , and A. Battaglia . 2023. “Exploring Stakeholders’ Interpretations of Safe Sport.” Journal of Sport & Social Issues 47, no. 1: 75–97. 10.1177/01937235221134610.

[ejsc70168-bib-0030] Hahn, H. P. 2023. “On the Changeful History of Franz Boas’s Concept of Cultural Relativism.” EAZ–Ethnographisch‐Archaeologische Zeitschrift 57, no. 1: 1–12. 10.54799/ISBF2790.

[ejsc70168-bib-0031] Hansen, P. Ø. , S. Chroni , E. A. Skille , and F. E. Abrahamsen . 2021. “Leading and Organising National Teams: Functions of Institutional Leadership.” Sports Coaching Review 10, no. 3: 274–294. 10.1080/21640629.2021.1896213.

[ejsc70168-bib-0032] Hartill, M. , B. Rulofs , M. Allroggen , et al. 2023. “Prevalence of Interpersonal Violence Against Children in Sport in Six European Countries.” Child Abuse & Neglect 146: 106513. 10.1016/j.chiabu.2023.106513. 37931542

[ejsc70168-bib-0033] Hartill, M. , D. Simonetti , P. E. Adami , et al. 2024. “Abuse and Violence in Sport: Italy General Report.” ChangeTheGame. https://www.changethegame.it/wp‐content/uploads/2024/11/Italy‐General‐Report‐CTG.pdf.

[ejsc70168-bib-0034] International Safeguarding Children in Sport Working Group . 2016. “International Safeguards for Children in Sport: A Guide for Organisations Who Work With Children.” https://www.end‐violence.org/sites/default/files/paragraphs/download/Implementation‐Guide‐for‐organisations‐who‐work‐with‐children‐A5‐version‐re.pdf.

[ejsc70168-bib-0035] Johansson, S. 2022. “From Policy to Practice: Measures Against Sexual Abuse by Swedish Sports Federations.” Frontiers in Sports and Active Living 4: 841653. 10.3389/fspor.2022.841653. 35308595 PMC8924436

[ejsc70168-bib-0036] Jones, R. L. , and C. Corsby . 2015. “A Case for Coach Garfinkel: Decision Making and what We Already Know.” Quest 67, no. 4: 439–449. 10.1080/00336297.2015.1082919.

[ejsc70168-bib-0037] Kassam, A. 2023. “‘Social Assassination’: Defiant Rubiales Refuses to Resign Over World Cup Kiss.” Guardian. https://www.theguardian.com/football/2023/aug/25/defiant‐rubiales‐bemoans‐social‐assassination‐and‐vows‐to‐stay‐as‐spanish‐fa‐head.

[ejsc70168-bib-0039] Kerr, G. , A. Battaglia , and A. Stirling . 2019. “Maltreatment in Youth Sport: A Systemic Issue.” Kinesiology Review 8, no. 3: 237–243. 10.1123/kr.2019-0016.

[ejsc70168-bib-0041] Khomutova, A. , S. Chroni , E. Kavanagh , et al. 2025. “FEPSAC Position Statement on Safeguarding Athletes in Sport.” Psychology of Sport and Exercise 80: 102897. 10.1016/j.psychsport.2025.102897. 40436228

[ejsc70168-bib-0042] Knoppers, A. , C. van Doodewaard , and R. Spaaij . 2024. “(Un) Doing Gender Inequalities in Sport Organizations.” Journal of Sport Management 38, no. 6: 438–446. 10.1123/jsm.2024-0032.

[ejsc70168-bib-0043] Kvale, S. , and S. Brinkmann . 2009. Interviews: Learning the Craft of Qualitative Research Interviewing. 2nd ed. Sage Publications.

[ejsc70168-bib-0044] Lang, M. , and M. Hartill , eds. 2014. Safeguarding, Child Protection and Abuse in Sport: International Perspectives in Research, Policy and Practice. Routledge.

[ejsc70168-bib-0045] McGee, S. , G. Kerr , M. Atkinson , and A. Stirling . 2024. ““I Always Just Viewed It as Part of Sport”: Psychological Maltreatment and Conformity to the Sport Ethic.” Journal of Applied Sport Psychology 37, no. 4: 1–20. 10.1080/10413200.2024.2414002.

[ejsc70168-bib-0046] McMahon, J. , C. Zehntner , K. R. McGannon , and M. Lang . 2020. “The fast‐tracking of One Elite Athlete Swimmer into a Swimming Coaching Role: A Practice Contributing to the Perpetuation and Recycling of Abuse in Sport?” European Journal for Sport and Society 17, no. 3: 265–284. 10.1080/16138171.2020.1792076.

[ejsc70168-bib-0047] Mountjoy, M. , C. Brackenridge , M. Arrington , et al. 2016. “International Olympic Committee Consensus Statement: Harassment and Abuse (Non‐Accidental Violence) in Sport.” British Journal of Sports Medicine 50, no. 17: 1019–1029. 10.1136/bjsports-2016-096121. 27118273

[ejsc70168-bib-0048] Muscat, A. , M. Nesti , D. Richardson , and M. Littlewood . 2020. “Small Island Mentality: Migratory Experiences of Elite Level Footballers.” International Journal of Sports, Health and Physical Education 2, no. 1: 28–37. 10.33545/26647559.2020.v2.i1a.19.

[ejsc70168-bib-0049] Nite, C. , and J. Nauright . 2020. “Examining Institutional Work That Perpetuates Abuse in Sport Organizations.” Sport Management Review 23, no. 1: 117–129. 10.1016/j.smr.2019.06.002.

[ejsc70168-bib-0050] Norman, L. 2010. “Feeling Second Best: Elite Women Coaches’ Experiences.” Sociology of Sport Journal 27, no. 1: 89–104. 10.1123/ssj.27.1.89.

[ejsc70168-bib-0051] Ohlert, J. , H. Schmitz , A. Schäfer‐Pels , and M. Allroggen . 2022. “An Empowering Climate as a Protective Factor Against Sexual Violence in Sport?” Social Sciences 11, no. 8: 330. 10.3390/socsci11080330.

[ejsc70168-bib-0052] Ohlert, J. , T. Vertommen , B. Rulofs , T. Rau , and M. Allroggen . 2021. “Elite Athletes’ Experiences of Interpersonal Violence in Organized Sport in Germany, the Netherlands, and Belgium.” European Journal of Sport Science 21, no. 4: 604–613. 10.1080/17461391.2020.1781266. 32524909

[ejsc70168-bib-0053] Parent, S. , and M. P. Vaillancourt‐Morel . 2021. “Magnitude and Risk Factors for Interpersonal Violence Experienced by Canadian Teenagers in the Sport Context.” Journal of Sport & Social Issues 45, no. 6: 528–544. 10.1177/0193723520973571.

[ejsc70168-bib-0054] Patton, M. Q. 2015. Qualitative Research & Evaluation Methods: Integrating Theory and Practice. 4th ed. Sage publications.

[ejsc70168-bib-0055] Reher, D. S. 1998. “Family Ties in Western Europe: Persistent Contrasts.” Population and Development Review 24, no. 2: 203–234. 10.2307/2807972.

[ejsc70168-bib-0056] Rhind, D. J. , and F. Owusu‐Sekyere . 2020. “Evaluating the Impacts of Working Towards the International Safeguards for Children in Sport.” Sport Management Review 23, no. 1: 104–116. 10.1016/j.smr.2019.05.009.

[ejsc70168-bib-0057] Roberts, V. , V. Sojo , and F. Grant . 2020. “Organisational Factors and Non‐Accidental Violence in Sport: A Systematic Review.” Sport Management Review 23, no. 1: 8–27. 10.1016/j.smr.2019.03.001.

[ejsc70168-bib-0058] Rulofs, B. , and M. Hartill . 2025. “Child Abuse in Sport: Critical perspectives—An Introduction.” In Child Abuse in Sport, edited by M. Hartill and B. Rulofs , 1–5. Emerald Publishing Limited. 10.1108/S1476-285420250000025001.

[ejsc70168-bib-0059] Rulofs, B. , M. D. Topič , R. Diketmüller , et al. 2019. “Final Report Voices for Truth and Dignity: Combatting Sexual Violence in European Sport Through the Voices of Those Affected.” http://voicesfortruthanddignity.eu/wp‐content/uploads/2020/02/VOICE_Final‐Report_kompr.pdf.

[ejsc70168-bib-0060] Ryba, T. V. 2017. “Cultural Sport Psychology: A Critical Review of Empirical Advances.” Current Opinion in Psychology 16: 123–127. 10.1016/j.copsyc.2017.05.003. 28813335

[ejsc70168-bib-0061] Ryba, T. V. , and H. K. Wright . 2005. “From Mental Game to Cultural Praxis: A Cultural Studies Model’s Implications for the Future of Sport Psychology.” Quest 57, no. 2: 192–212. 10.1080/00336297.2005.10491853.

[ejsc70168-bib-0063] Schmidt, R. E. , A. R. Schneeberger , and M. C. Claussen . 2022. “Interpersonal Violence Against Athletes.” Sports Psychiatry 1, no. 2: 78–84. 10.1024/2674-0052/a000014.

[ejsc70168-bib-0064] Seanor, M. E. , C. E. Giffin , R. J. Schinke , D. A. Coholic , and M. Larivière . 2023. “Pixies in a Windstorm: Tracing Australian Gymnasts’ Stories of Athlete Maltreatment Through Media Data.” Sport in Society 26, no. 3: 553–572. 10.1080/17430437.2022.2060825.

[ejsc70168-bib-0065] Silverman, D. 2021. Doing Qualitative Research. Sage Publications.

[ejsc70168-bib-0066] Skille, E. , P. Ø. Hansen , F. Abrahamsen , and S. Chroni . 2020. “National and Organizational Culture in Norwegian Elite Sport: The Account of National Handball Head Coaches.” Scandinavian Sport Studies Forum 11: 93–116. https://idrottsforum.org/skilleetal201021/.

[ejsc70168-bib-0067] Skille, E. Å. , and S. Chroni . 2018. “Norwegian Sports Federations’ Organizational Culture and National Team Success.” International Journal of Sport Policy and Politics 10, no. 2: 321–333. 10.1080/19406940.2018.1425733.

[ejsc70168-bib-0068] Smith, B. , and K. R. McGannon . 2018. “Developing Rigor in Qualitative Research: Problems and Opportunities Within Sport and Exercise Psychology.” International Review of Sport and Exercise Psychology 11, no. 1: 101–121. 10.1080/1750984X.2017.1317357.

[ejsc70168-bib-0069] Snape, J. , and A. Kassam . 2023. “Spanish Football President’s Kiss Sparks Outrage After Women’s World Cup Final.” Guardian. https://www.theguardian.com/football/2023/aug/21/luis‐rubiales‐kiss‐outrage‐spanish‐football‐fa‐president‐womens‐world‐cup‐final‐spain‐jenni‐hermoso.

[ejsc70168-bib-0070] Stirling, A. E. , and G. A. Kerr . 2009. “Abused Athletes’ Perceptions of the Coach‐Athlete Relationship.” Sport in Society 12, no. 2: 227–239. 10.1080/17430430802591019.

[ejsc70168-bib-0071] Tuakli‐Wosornu, Y. A. , K. Burrows , K. Fasting , et al. 2024. “IOC Consensus Statement: Interpersonal Violence and Safeguarding in Sport.” British Journal of Sports Medicine 58, no. 22: 1322–1344. 10.1136/bjsports-2024-108766. 39586634

[ejsc70168-bib-0072] Vertinsky, P. 2012. “Is There a “Beyond Patriarchy” in Feminist Sport History?” Journal of Sport History 39, no. 3: 479–486. 10.5406/jsporthistory.39.3.479.

[ejsc70168-bib-0073] Vertommen, T. , N. Schipper‐van Veldhoven , K. Wouters , et al. 2016. “Interpersonal Violence Against Children in Sport in the Netherlands and Belgium.” Child Abuse & Neglect 51: 223–236. 10.1016/j.chiabu.2015.10.006. 26516053

[ejsc70168-bib-0074] Wilson, O. W. , C. Whatman , S. Walters , et al. 2022. “The Value of Sport: Wellbeing Benefits of Sport Participation During Adolescence.” International Journal of Environmental Research and Public Health 19, no. 14: 8579. 10.3390/ijerph19148579. 35886430 PMC9324252

[ejsc70168-bib-0075] World Health Organization (WHO) . 2002. World Report on Violence and Health 2002. World Health Organization. https://www.who.int/publications/i/item/9241545615.

[ejsc70168-bib-0076] World Health Organization (WHO) . 2014. Global Status Report on Violence Prevention 2014. World Health Organization. https://www.who.int/publications/i/item/9789241564793.

[ejsc70168-bib-0077] World Health Organization (WHO) . 2020. Global Status Report on Preventing Violence Against Children 2020 2014. World Health Organization. https://apps.who.int/iris/handle/10665/332394.

